# Haustorium formation and a distinct biotrophic transcriptome characterize infection of *Nicotiana benthamiana* by the tree pathogen *Phytophthora kernoviae*

**DOI:** 10.1111/mpp.13072

**Published:** 2021-05-20

**Authors:** Shumei Wang, Ramesh R. Vetukuri, Sandeep K. Kushwaha, Pete E. Hedley, Jenny Morris, David J. Studholme, Lydia R. J. Welsh, Petra C. Boevink, Paul R. J. Birch, Stephen C. Whisson

**Affiliations:** ^1^ Division of Plant Sciences University of Dundee James Hutton Institute Invergowrie, Dundee UK; ^2^ Department of Plant Breeding Swedish University of Agricultural Sciences Alnarp Sweden; ^3^ National Institute of Animal Biotechnology Hyderabad India; ^4^ Cell and Molecular Sciences James Hutton Institute Invergowrie, Dundee UK; ^5^ Biosciences, College of Life and Environmental Sciences University of Exeter Exeter UK

**Keywords:** biotrophy, effector, pathogenicity, RNA‐Seq, RXLR, tree disease

## Abstract

*Phytophthora* species cause some of the most serious diseases of trees and threaten forests in many parts of the world. Despite the generation of genome sequence assemblies for over 10 tree‐pathogenic *Phytophthora* species and improved detection methods, there are many gaps in our knowledge of how these pathogens interact with their hosts. To facilitate cell biology studies of the infection cycle we examined whether the tree pathogen *Phytophthora kernoviae* could infect the model plant *Nicotiana benthamiana*. We transformed *P. kernoviae* to express green fluorescent protein (GFP) and demonstrated that it forms haustoria within infected *N. benthamiana* cells. Haustoria were also formed in infected cells of natural hosts, *Rhododendron ponticum* and European beech (*Fagus sylvatica*). We analysed the transcriptome of *P. kernoviae* in cultured mycelia, spores, and during infection of *N. benthamiana*, and detected 12,559 transcripts. Of these, 1,052 were predicted to encode secreted proteins, some of which may function as effectors to facilitate disease development. From these, we identified 87 expressed candidate RXLR (Arg‐any amino acid‐Leu‐Arg) effectors. We transiently expressed 12 of these as GFP fusions in *N. benthamiana* leaves and demonstrated that nine significantly enhanced *P. kernoviae* disease progression and diversely localized to the cytoplasm, nucleus, nucleolus, and plasma membrane. Our results show that *N. benthamiana* can be used as a model host plant for studying this tree pathogen, and that the interaction likely involves suppression of host immune responses by RXLR effectors. These results establish a platform to expand the understanding of *Phytophthora* tree diseases.

## Graphical abstract

Tree pathogen *Phytophthora kernoviae* forms digit‐shaped haustoria during infection of model and natural hosts, and secretes RXLR effectors that promote infection and localize to diverse cellular locations.

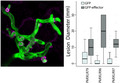

## 1 INTRODUCTION

Trees are long‐lived organisms that must contend with prolonged exposure to attack by multiple pathogens. Some diseases of trees are particularly prominent in public awareness, such as Dutch elm disease, chestnut blight, ash dieback, and sudden oak death, caused by *Ophiostoma novo‐ulmi*, *Cryphonectria parasitica*, *Hymenoscyphus fraxineus*, and *Phytophthora ramorum*, respectively.

*Phytophthora* species are prevalent among the most damaging tree pathogens and are prominent in lists of emerging biosecurity threats globally (Brasier, 2008; Hyun & Choi, 2014; Santini et al., 2013). One such species is *Phytophthora kernoviae*, which has a broad host range and can cause disease on forest trees, understorey plants, and invasive and ornamental plant species (Beales et al., 2009; Brasier et al., 2005; Fichtner et al., 2012; Gardner et al., 2015; Sanfuentes et al., 2016). The host range of *P. kernoviae* overlaps with other tree‐infecting species such as *P. ramorum* and *P. pseudosyringae* (Beales et al., 2010; Jung et al., 2003; Martin & Tooley, 2003). Although first identified in the UK, *P. kernoviae* has also been found in the Republic of Ireland, New Zealand, and Chile, and may originate in New Zealand (Gardner et al., 2015; Sanfuentes et al., 2016; Studholme et al., 2019). There is concern both within the UK and internationally regarding the potential spread of *P. kernoviae* (Drake & Jones, 2017; Fichtner et al., 2012; Hyun & Choi, 2014; Tracy, 2009; Widmer, 2015). Most research on this species has been directed at understanding its potential host range, epidemiology, and improving detection methods (Gardner et al., 2015; Kong et al., 2012; Miles et al., 2015; Mulholland et al., 2015; Schwenkbier et al., 2015; Widmer, 2015).

The *Phytophthora* genus comprises over 140 species organized into 12 clades (Jung et al., 2017; Yang et al., 2017). Tree‐infecting species are found in all clades of the genus such as *P. plurivora* in Clade 2, *P. pseudosyringae* in Clade 3, *P. cinnamomi* in Clade 7, and *P. ramorum* in Clade 8. *P. kernoviae* is more distantly related to these species, in Clade 10, along with *P. boehmeriae*, *P. morindae*, *P. gondwanensis*, *P. gallica*, and *P. intercalaris* (Yang et al., 2017).

*P. kernoviae* causes disease and sporulates on *Rhododendron* leaves, and causes bleeding stem cankers on a range of tree species where it may spread via the xylem or establish asymptomatic infections in roots and leaves (Brasier et al., 2005; Brown & Brasier, 2007; Denman et al., 2009; Fichtner et al., 2011). That *P. kernoviae* can establish asymptomatic infections suggests that it either evades detection or actively forms a biotrophic interaction with its host plants.

Although there are draft genome assemblies for *P. kernoviae* strains from diverse geographical sites (Feau et al., 2016; Sambles et al., 2015; Studholme et al., 2016, 2019), these resources remain to be fully exploited to advance understanding of the biology of this species. Like other *Phytophthora* species, the *P. kernoviae* genome encodes numerous candidate effector proteins (McGowan & Fitzpatrick, 2017) that may act to facilitate infection through a variety of mechanisms (Wang, Tyler, et al., 2019). Prominent among effector proteins in *Phytophthora* species are those that possess an N‐terminal signal peptide for secretion from the pathogen cell and an RXLR peptide motif (Arg‐any amino acid‐Leu‐Arg) located near the N‐terminus of the protein. The majority of RXLR effectors studied to date also possess an EER motif (Glu‐Glu‐Arg) downstream of the RXLR (Whisson et al., 2016). In *P. infestans* and *P. sojae*, the RXLR‐EER region directs effector translocation from haustoria into host plant cells (Dou et al., 2008; Whisson et al., 2007). Haustoria are pathogen structures that project into plant cells from the invading intercellular hyphae and are bounded by the host cell plasma membrane. *Phytophthora* haustoria are the sites of secretion for diverse effector proteins delivered by at least two different secretion pathways (Kagda et al., 2020; Liu et al., 2014; Meng et al., 2015; Wang et al., 2017, 2018).

For *P. kernoviae* and other tree‐infecting *Phytophthora* spp., there are many questions to be addressed regarding how these pathogens interact with host plants. This ranges from how different tissues are colonized, whether haustoria are formed, whether interactions are biotrophic or necrotrophic, through to whether RXLR effector proteins are used to facilitate host infection. Examining pathogen growth within large woody host plants that may have strictly seasonal patterns of growth is difficult. Host plants with shorter generation times and that are more amenable to laboratory analyses would help to alleviate this. The model plant *Nicotiana benthamiana* has been used as a tractable experimental host to study the cell biology of plant–*Phytophthora* interactions (Boevink et al., 2020) and we show here that it is also suitable for studies of *P. kernoviae*. We made stable transformed lines of *P. kernoviae* expressing green fluorescent protein (GFP) and used them to identify haustoria formation in leaves of *N. benthamiana*, *Rhododendron*, and European beech. Transcriptome analysis of *P. kernoviae* infection demonstrated expression of numerous RXLR effectors. When expressed in host tissue, a subset of these effectors was shown to localize to different subcellular sites and promote *P. kernoviae* infection.

## 2 RESULTS

### 2.1 *P. kernoviae* can infect the model plant *N. benthamiana*

**FIGURE 1:**
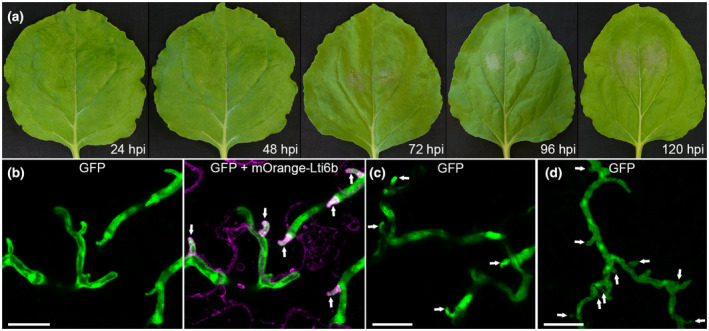
*Phytophthora* *kernoviae* infection progression in model host *Nicotiana benthamiana*, and natural hosts beech and rhododendron. (a) *N*. *benthamiana* leaves imaged at (L–R) 24 hours postinoculation (hpi) with wild‐type *P. kernoviae*, 48, 72, 96, and 120 hpi as indicated. Macroscopic necrosis symptoms were not visible until 72 hpi. (b) Confocal projection image showing that green fluorescent protein (GFP)‐labelled *P. kernoviae* forms finger‐like haustoria during infection of transgenic *N. benthamiana*, with plasma membrane labelled by an mOrange‐Lti6b fusion (shown in magenta). Haustoria were surrounded by labelled plant plasma membrane, indicating penetration of the plant cell wall and invagination of the plasma membrane. (c, d) Confocal imaging of natural hosts rhododendron (c) and beech (d) leaf tissue infected by the same transformant used in (b) revealed that *P. kernoviae* appeared to form haustoria in woody host plants. Haustoria are indicated by arrows. Scale bars represent 20 µm

Inoculated *N. benthamiana* leaves remained green and symptomless for the first 48 hr postinoculation (hpi). The first disease symptoms were visible by 72 hpi as necrotic areas surrounding inoculation sites (Figure 1a). These observations suggest that *P. kernoviae* has a hemibiotrophic interaction with *N. benthamiana*, where infected tissue remains alive for up to 48 hpi before infection leads to host cell death.

We generated 13 transgenic *P. kernoviae* lines to constitutively express GFP, of which one (PkGFP8) expressed GFP at a high level. PkGFP8 growth in culture was indistinguishable from the nontransgenic progenitor and readily infected *N. benthamiana*, so was used for confocal microscopy analyses. Infections of *N. benthamiana* leaves with PkGFP8 revealed infection stages typical of *Phytophthora* species (Boevink et al., 2020; Hardham, 2007): host invasion and initial hyphal colonization by 24 hpi, followed by extensive hyphal spread by 48 hpi, before macroscopic symptoms were visible. Digit‐like projections were observed on hyphae ramifying through the leaf tissue. To determine if these projections were haustoria, we infected transgenic *N. benthamiana* plants stably expressing an mOrange‐LTi6b fusion that labels the plant plasma membrane (Kurup et al., 2005). This revealed that the fluorescently labelled plasma membrane surrounded the hyphal projections and, as such, they are likely to be biotrophic haustoria (Figure 1b).

PkGFP8 was also used to infect leaves of *Rhododendron ponticum* and *Fagus sylvatica* (beech). A fluorescently labelled plasma membrane marker was not available in these hosts, but imaging of presymptomatic leaf tissue revealed haustoria on the invading hyphae of *P. kernoviae* (Figure 1c,d). This suggests that the interaction with host tissue in *N. benthamiana* is similar to that in natural hosts *Rhododendron* and beech, and indicates that *N. benthamiana* is a useful model host to study *P. kernoviae* pathogenicity.

### 2.2 The *P. kernoviae* transcriptome reveals effectors expressed during early infection stages

The *P. kernoviae* transcriptome was explored by RNA sequencing (RNA‐Seq) of cultured mycelium, mixed asexual spores (sporangia and zoospores), and three different infection stages in *N*. *benthamiana* leaves (24, 48, and 72 hpi). Illumina sequencing generated 1,206 million raw reads. After removal of low‐quality and contaminant reads, and trimming, a total of 1,102 million reads remained, yielding an average of 55 million reads per sample (Table S1). An average of 86% of the reads from mycelium and spore libraries were successfully mapped to the *P. kernoviae* reference genome sequence. The proportion of reads from *P. kernoviae* in the infection samples ranged from an average of 0.3% (24 hpi) to 2.5% (72 hpi), which was supplemented by increased sequencing depth for infection samples (average 65 million reads).

All sequence reads were initially assembled into 12,559 transcripts (Table S2). We performed principal component analysis (PCA) of the sample correlation matrix calculated from log_2_‐transformed normalized expression count values from DESeq2 (Figure S1a). The first principal component comprises 81% of the total variance between samples, while the next principal component accounted for 11% of overall variance. Hierarchical clustering revealed that sample classes typically clustered together except T24_rep2, which differed from other samples (Figure S1b). Analysis of differentially expressed *P. kernoviae* genes (DEGs) between the five samples revealed 8,065 differentially expressed transcripts with a log_2_‐fold change ≥2.0 and a false discovery rate (FDR) of <0.05 (Tables S3 and S4).

**FIGURE 2:**
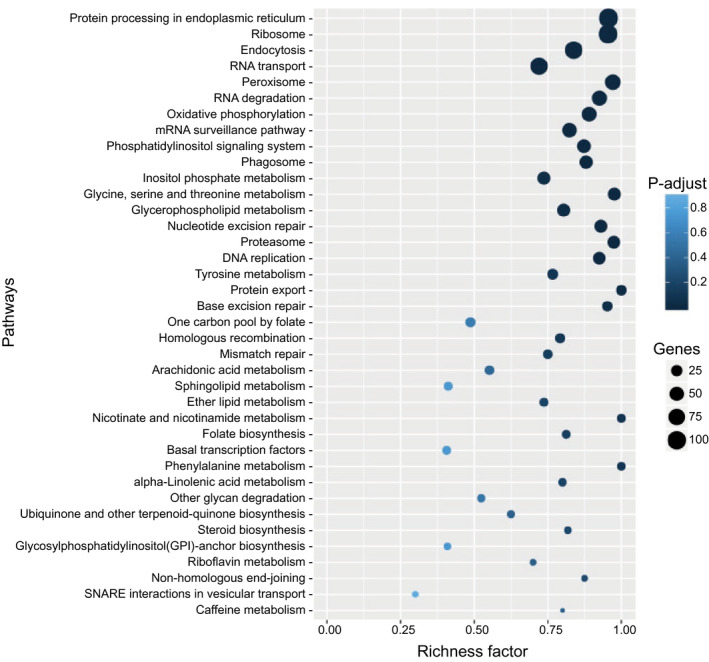
KEGG pathway enrichment for *Phytophthora* *kernoviae* differentially expressed genes (DEGs). The richness factor indicates the ratio of numbers of DEGs annotated for a specific pathway term, compared to the number of all genes annotated for that pathway. The size of the circles represents the number of DEGs annotated for that pathway or process

Volcano plot analysis (filtering on both significance and fold‐change) and comparison of DEGs at different infection stages revealed that the infection stages had the least number of DEGs when compared against each other, while comparisons of infection stages with in vitro cultured mycelium revealed at least six‐fold more DEGs (Table 1 and Figure S1c). The greatest number of DEGs was identified when comparing in vitro cultured mycelia with spores (Table 1), probably due in part to the spore samples comprising mixed transcriptomes of both sporangia and zoospores. We identified 60 differentially expressed candidate RXLR effectors and 164 carbohydrate active proteins (CAZymes) (Tables S5 and S6). Mapping DEGs onto pathways revealed the greatest number of genes were involved in core cellular processes such as protein processing, ribosome function, endocytosis, and RNA transport (Figure 2). Metabolic pathways most enriched for DEGs were oxidative phosphorylation, inositol phosphate metabolism, glycine, serine and threonine metabolism, glycerophospholipid metabolism, and tyrosine metabolism.

**TABLE 1:** Comparison of the number of differentially expressed genes in *Phytophthora kernoviae* during infection of *Nicotiana benthamiana* and in culture (log_2_‐fold change ≥2.0, false discovery rate of <0.05)

	24 hpi	48 hpi	72 hpi	Spores	Mycelium
24 hpi		76	194	2,609	1,666
48 hpi			152	4,606	1,133
72 hpi				4,101	1,290
Spores					5,917
Mycelium					

hpi, hours postinoculation.

The most abundant transcripts in all samples typically encompassed genes encoding ribosomal proteins, but with some additional genes, depending on the samples (Table S2). In cultured mycelium, two of the most strongly expressed genes encoded glycolytic enzymes glyceraldehyde‐3‐phosphate dehydrogenase (TCONS_00003986.p1) and fructose bisphosphate aldolase (TCONS_00007488.p1). In spore samples, the gene encoding CDC14 phosphatase (TCONS_00005250.p1), involved in sporangium formation in other *Phytophthora* species (Ah Fong & Judelson, 2003; Zhao et al., 2011), was the most highly expressed functionally annotated gene (Table S2). A homolog of the *Phytophthora* cell wall transglutaminase (TCONS_00009334.p1), an elicitor of plant defence responses (Brunner et al., 2002), was also highly expressed in spore samples, although this *P. kernoviae* homolog does not possess an intact PEP13 elicitor sequence. At 24 hpi, a gene encoding heat shock protein 70 (Hsp70) (TCONS_00007412.p1) was the most highly expressed, while at 72 hpi it was a gene encoding ATP synthase (TCONS_00008180.p1).

#### 2.2.1 Cutinase

Transcripts for two cutinases were identified in *P. kernoviae*: TCONS_00002107.p4 and TCONS_00005046.p1. Transcripts for TCONS_00005046.p1 were more readily detected than TCONS_00002107.p4, reaching peak expression at 24 hpi (Figure S2 and Table S6), suggesting a role in breaching the plant cuticle.

#### 2.2.2 Carbohydrate active enzymes

Transcripts were identified for 211 candidate carbohydrate active enzymes (CAZymes) potentially involved in degrading polysaccharides or auxiliary activities (Figure S2 and Table S6). These encompass 31 glycoside hydrolase (GH) families, four polysaccharide lyase (PL) families, six carbohydrate esterase (CE) families, and six auxiliary activity (AA) families (Table S6).

To approximate the expression for each CAZy protein family identified, the expression values were summed for each family (Table S7), as performed previously for *P. infestans* and *P. parasitica* (Ah Fong, Kim, et al., 2017; Ah Fong, Shrivastava, et al., 2017; Blackman et al., 2015). This revealed that CAZy family GH17 (β‐1,3‐glucosidase) was among the most highly expressed in all *P. kernoviae* samples. In addition to GH17 expression, in cultured mycelium the most highly expressed CAZy families were GH30_1 and GH16, comprising glucosylceramidase and β‐glucan synthesis‐associated activities, respectively. In spores, the most highly expressed CAZy families were families GH6 and GH5_20, both comprising endo‐β‐1,4‐glucanase (cellulase) activity. At both 24 and 48 hpi, the most highly expressed CAZy families were families AA2 and AA7 comprising haem‐peroxidase and glucooligosaccharide oxidase (annotated as berberine‐like) activities. At 72 hpi, families GH3 and GH1 were the most highly expressed, both comprising β‐glucosidase activity. When comparing CAZy family level expression in cultured mycelium to infection stages, the most highly up‐regulated CAZy families were those that potentially act on plant polysaccharides such as cellulose (GH7, GH1, GH131), hemicellulose (GH12), starch (GH31), and pectins (GH43_6, GH78, PL3_2) (Table S7).

#### 2.2.3 Secreted apoplastic proteins that interact with host cells to influence infection

From the *P. kernoviae* transcripts detected, 1,052 were predicted to encode secreted proteins without a predicted transmembrane domain (Table S2), including homologs of microbe‐associated molecular patterns (MAMPs) or apoplastic effectors in other *Phytophthora* species (Table S8). Elicitins, ubiquitous secreted proteins from many *Phytophthora* species well known as MAMPs (Raaymakers & van den Ackerveken, 2016), were represented by 34 transcripts in *P. kernoviae*. Transcripts for eight elicitins were present at higher levels in cultured mycelium than in spores or during infection. Two elicitins (TCONS_00005991.p1, TCONS_00000289.p7) were expressed at very low levels in cultured mycelium, but markedly up‐regulated by 72 hpi (Table S8).

*Phytophthora* species possess numerous secreted necrosis and ethylene‐inducing peptide 1‐like (NLP) proteins that may be recognized as MAMPs (McGowan & Fitzpatrick, 2017; Raaymakers & van den Ackerveken, 2016). We identified 20 candidate NLPs in the *P. kernoviae* transcriptome, with seven of these exhibiting up‐regulation during infection (Table S8). Of these, three exhibited greatest transcript accumulation at 72 hpi (TCONS_00004814.p1, TCONS_00009318.p1, TCONS_00006796.p1), three at 48 hpi (TCONS_00005503.p1, TCONS_00007512.p1, TCONS_00005701.p1), and one at 24 hpi (TCONS_00001592.p1).

*P. kernoviae* expresses an ortholog of haustorium‐specific membrane protein Hmp1 (TCONS_00003541.p1), first described from *P. infestans* (Avrova et al., 2008). As in *P. infestans*, transcripts encoding this protein were most abundant before disease symptoms were visible (48 hpi; Table S8).

*P. kernoviae* expressed nine genes encoding predicted Kazal domain proteins (Table S8), which may inhibit apoplastic serine proteases (Tian et al., 2004, 2005). With the exceptions of TCONS_00005697.p2 and TCONS_00006350.p1, peak transcript accumulation for these genes occurred during infection. TCONS_00005245.p1 exhibited strong up‐regulation and high expression during infection. Expression of two candidate cysteine protease inhibitors was also detected in *P. kernoviae*, but neither exhibited up‐regulation during infection (Table S8).

*P. infestans* and *P. sojae* secrete glucanase inhibitor proteins (GIPs) to counter host β‐1,3‐glucanases (Damasceno et al., 2008; Rose et al., 2002). *P. kernoviae* expresses a GIP1 homolog (TCONS_00006575.p1) that showed strongly elevated transcript levels by 48 hpi, with transcript levels decreasing by 72 hpi (Table S8).

Secreted carbonic anhydrases and berberine proteins have been proposed as effectors in *P. infestans* and other *Phytophthora* species (Hosseini et al., 2015; Raffaele et al., 2010). Transcripts for three candidate berberine proteins accumulated to high levels during infection (Table S6). BLASTP searches of NCBI GenBank revealed that these were equally likely to represent glucooligosaccharide oxidases and are described in the CAZymes section, family AA7. Transcripts for nine candidate carbonic anhydrases were identified, of which three (TCONS_00008455.p1, TCONS_00003845.p1, TCONS_00004372.p2) were up‐regulated during infection (Table S8). The most highly expressed of these was TCONS_00003845, which exhibited greatest transcript levels at 24 hpi.

#### 2.2.4 Crinkle and necrosis effectors

We identified 12 transcripts encoding proteins with an LXLFLAK motif characteristic of candidate crinkle and necrosis (CRN) effectors (Amaro et al., 2017) (Table S9). None of these possess a predicted signal peptide for secretion. Two candidate CRN proteins had average normalized expression counts <1, nine exhibited maximum transcript levels in spore samples, and one (TCONS_00003091.p8) reached peak expression during infection at 24 hpi. Candidate CRN effectors exhibiting the highest transcript levels (in spores) were TCONS_00009308.p1, TCONS_00007157.p1, and TCONS_00007408.p1; all three were also expressed strongly throughout infection.

#### 2.2.5 RXLR effectors

**FIGURE 3:**
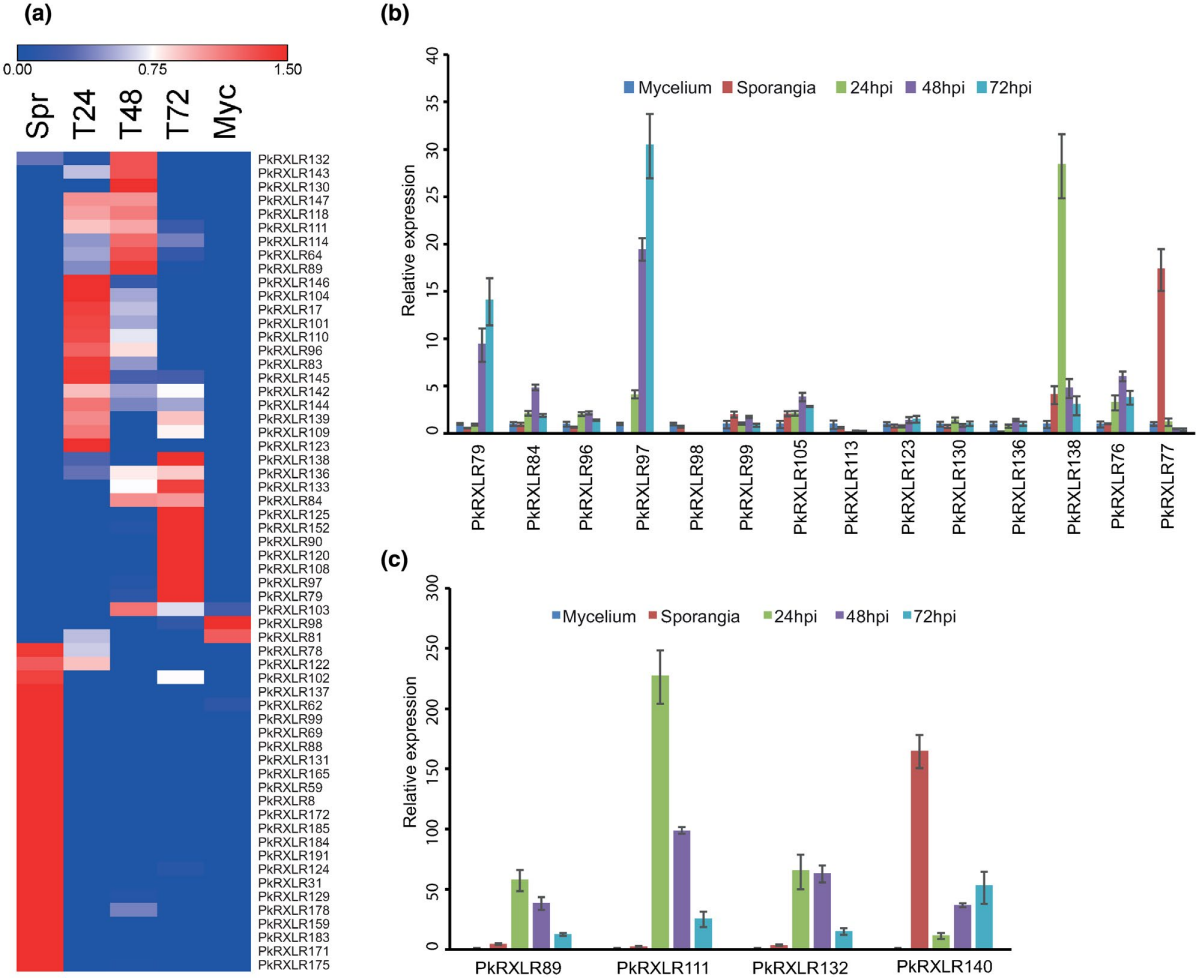
Expression of RXLR effector‐encoding genes in *Phytophthora* *kernoviae*. (a) Heatmap of differentially expressed RXLR effector genes (RNA sequencing) in cultured mycelium (Myc), spores (mixture of sporangia and zoospores; Spr), and infection of *Nicotiana benthamiana* at 24, 48, and 72 hr postinoculation (hpi) (T24, T48, T72). Red shading indicates increased transcript levels, and blue shading indicates lower transcript levels. Colour shading represents expression values transformed by subtracting the mean and dividing by the standard deviation for each row (gene). (b, c) The relative expression of 18 predicted RXLR effectors in *P. kernoviae* was validated by quantitative reverse transcription PCR. Transcript levels were compared to cultured mycelium, which was normalized to a value of 1. Error bars shown are standard error. Two additional independent biological replications are shown in Figure S3

We identified 193 candidate RXLR‐EER effectors from *P. kernoviae* genomic sequences (Table S10); candidates with/without predicted signal peptides were included to allow for mispredicted gene start codons or missing 5′ ends. Transcripts encoding 87 were detected, with 84 having average normalized expression counts >1 in at least one sample type. Transcripts encoding 69 RXLR effectors were detected with an expression count >1 during infection of *N. benthamiana*, of which 52 were differentially expressed (log_2_ fold‐change >2) (Figure 3 and Tables S3, S4, and S5). Two of the highest expressed candidate RXLR genes may be false‐positive predictions, as PkRXLR153 exhibits BLASTP similarity (6e^−95^) to ribosomal protein L15, while PkRXLR151 is similar to transposable elements (e 0.0); neither are differentially expressed. Further highly expressed candidate RXLR effectors were PkRXLR147, PkRXLR114, PkRXLR152, PkRXLR104, PkRXLR96, and PkRXLR143 (Table S5). Peak in planta expression of 25 RXLR effectors occurred at 24 hpi, and a further 23 reached peak in planta expression at 48 hpi before macroscopic disease symptoms were visible.

Quantitative reverse transcription polymerase chain reaction (RT‐qPCR) analysis was used to validate expression of 18 RXLR effectors during *P. kernoviae* infection of *N. benthamiana*. These were selected across the range of expression levels found in RNA‐Seq, including some that were not detected. Expression profiles from three independent biological replicates, which were additional to the samples used for RNA‐Seq, were in broad agreement with the RNA‐Seq data. Compared to expression in cultured mycelium, RXLR effectors PkRXLR79, 89, 97, 111, 132, and 138 exhibited the greatest relative up‐regulation during plant infection, ranging from 14‐ to 350‐fold (Figures 3 and S3). Other RXLR effector‐encoding genes exhibited lower levels of up‐regulation, or none, in some replicates (e.g., PkRXLR96, 98, and 99). An exception was PkRXLR77, which was up‐regulated up to 20‐fold in spores.

### 2.3 In planta expression of selected RXLR effectors enhanced *P. kernoviae* colonization of *N. benthamiana* leaves

**FIGURE 4:**
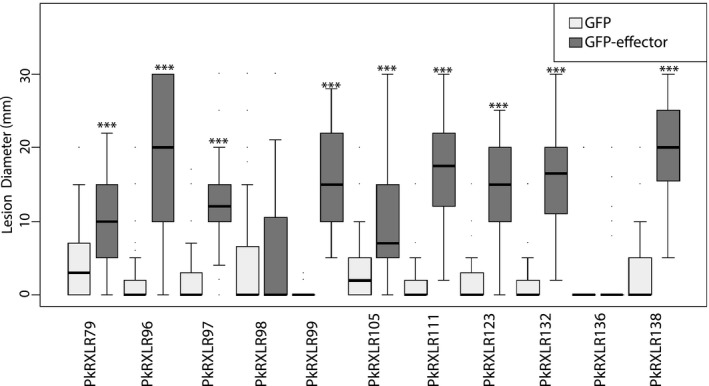
Candidate RXLR effectors from *Phytophthora* *kernoviae* can enhance colonization of *Nicotiana benthamiana*. N‐terminally green fluorescent protein (GFP)‐tagged candidate effectors were transiently and individually expressed in one half of *N*. *benthamiana* leaves, while free GFP was expressed on the other half of each leaf as control. The diameters (mm) of macroscopically visible disease lesions were measured at 4 days postinoculation (dpi). Nine of the 12 tested effectors significantly boosted colonization by *P. kernoviae* compared to the GFP control (indicated with asterisks). Effector PkRXLR89 caused cell death and is not shown here. *p* values were evaluated using the Student–Newman–Keuls method (*p < .05, **p < .01, ***p ≤ .001)

Previous studies have used transient expression of RXLR effectors inside plant cells, delivered by *Agrobacterium tumefaciens*, followed by pathogen infection (*A. tumefaciens* transient assay, ATTA; King et al., 2014; Qiao et al., 2013; Wang, McLellan, et al., 2019). In these assays, an increase in disease lesion diameter relative to an expressed GFP control suggests that the effector acts to facilitate infection. We selected nine *P. kernoviae* effectors that were observed to be up‐regulated during infection, compared to spores, in either RNA‐Seq or RT‐qPCR (PkRXLR79, 89, 96, 97, 98, 111, 123, 132, 136, and 138), and two that were not significantly differentially expressed (PkRXLR99 and 105) for ATTAs. PkRXLR89 caused a rapid cell death response in *N. benthamiana* and was not considered further. Two effectors, PkRXLR98 and 136, did not enhance infection. The remaining nine effectors all significantly enhanced *P. kernoviae* infection when expressed in *N. benthamiana* (Figure 4).

### 2.4 Localization of *P. kernoviae* RXLR effectors inside plant cells

**FIGURE 5:**
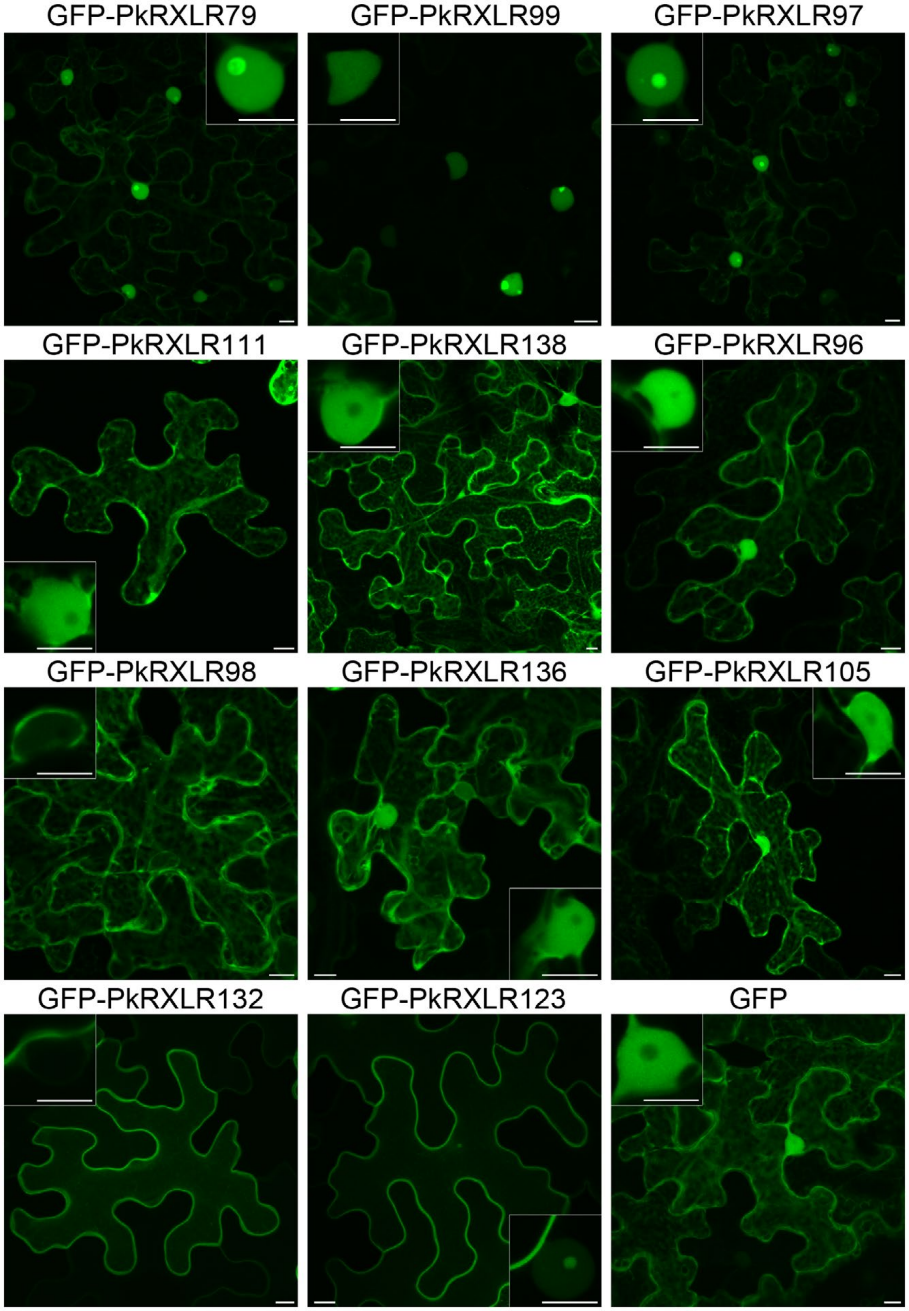
*Phytophthora* *kernoviae* RXLR effector‐GFP fusions localize to various compartments in plant cells. Confocal projection images of *Nicotiana benthamiana* cells expressing low levels of green fluorescent protein (GFP)‐effector fusions at 2 days after infiltration with *Agrobacterium tumefaciens* containing the expression vectors. Inset images are higher magnification single optical sections of the cell nuclei in the main images. GFP, not fused to an effector, showing nucleocytoplasmic localization, is included for comparison. Scale bars represent 10 µm

RXLR effectors translocate into plant cells (Wang et al., 2017, 2018) where they function at a variety of subcellular locations to promote infection (Caillaud et al., 2012; Wang, McLellan, et al., 2019). Confocal microscopy was used to localize the *P. kernoviae* N‐terminal GFP‐effector fusions used in ATTAs (previous section) in *N. benthamiana* leaf cells (Figure 5). A variety of localizations were observed, with effectors localized to the cytoplasm only (PkRXLR98), cytoplasm and nucleus (PkRXLR111, 138, 96, 136, and 105), nucleus only (PkRXLR99), nucleus and nucleolus (PkRXLR79 and 97), plasma membrane and nucleolus (PkRXLR123), and plasma membrane only (PkRXLR132). We attempted to localize RXLR effector PkRXLR89, which caused cell death in ATTA experiments. This effector formed protein aggregates in all experiments, even in cells with low levels of expression (not shown). Western blot analysis of the effectors expressed in *N. benthamiana* leaves showed that intact protein fusions were produced for all RXLR effectors tested (Figure S4).

Nuclear and nucleolar labelling observed for PkRXLR79, 99, and 97 was investigated further using known plant nuclear and nucleolar markers. Labelling the nucleoplasm with a coexpressed mRFP‐Histone2B (H2B) fusion showed that the GFP‐PkRXLR97 fusion uniformly labelled the nucleolus (Figure S5a). In contrast, GFP‐PkRXLR79 and 99 showed fluorescence in a ring surrounding the nucleolus in cells with low GFP‐effector expression (Figure S5a,b). Effector PkRXLR99 appeared to disrupt the nucleus even with low levels of expression, forming protein aggregates that excluded mRFP‐H2B fluorescence, but were separate to the nucleolus (Figure S5b).

Localization of GFP‐PkRXLR132 and 123 to the plasma membrane was confirmed by coexpression with the mOrange‐LTi6b fusion in transgenic *N. benthamiana* plants (Figure S6). Effector PkRXLR123 also exhibited labelling of the nucleus and nucleolus; the peptide sequence of this effector contains a predicted nuclear localization signal (Table S10).

## 3 DISCUSSION

*Phytophthora* species such as *P. kernoviae* cause some of the most damaging diseases of trees, and yet little is known of how they interact with host plants. Most research into *Phytophthora* pathogenesis and effector function is focused on crop infecting species. Like crop pathogenic species such as *P. infestans*, here we determine that *P. kernoviae* can form haustoria in infected plant tissue. This, plus the expression of RXLR effectors, suggests that, at least in the early stages of infection, it establishes a biotrophic interaction with host cells which necessitates suppression of plant immune responses by pathogen effectors.

Our finding that *P. kernoviae* could readily infect the model plant *N. benthamiana* overcame some of the challenges inherent with working with tree pathosystems. This model host enables transient gene expression or silencing, and noninvasive cell biology studies (Goodin et al., 2008). Furthermore, knowledge of *N. benthamiana* responses to *Phytophthora* infection (Whisson et al., 2016) enables the role of specific defence pathways and host targets of effectors to be readily determined for pathogens such as *P. kernoviae*.

*P. kernoviae* can be transformed and we generated GFP‐expressing strains, although only one of these was sufficiently fluorescent for confocal microscopy. This enabled us to investigate *P. kernoviae* infection development in *N. benthamiana* leaves, and natural hosts beech and *Rhododendron*. Like other *Phytophthora* species (Avrova et al., 2008; Boevink et al., 2020; Evangelisti et al., 2017; Wang et al., 2011, 2013), *P. kernoviae* forms digit‐like projections from intercellular hyphae during infection that are most likely to be haustoria because they are enveloped by the plant plasma membrane. *P. kernoviae* also appears to form haustoria in beech and *Rhododendron* leaves, indicating that this is a feature of its pathogenesis in natural hosts. In *P. infestans*, haustoria are major sites of protein secretion, and different classes of proteins involved in pathogenesis, such as CAZymes, protease inhibitors, elicitins, and RXLR effectors, are secreted from these structures (Kagda et al., 2020; Wang et al., 2017, 2018).

Our RNA‐Seq data provide the first insight into gene expression during infection for *P. kernoviae*, a tree pathogen more distantly related to other tree‐infecting species for which transcriptome analyses have been carried out during plant infection (Blackman et al., 2015; Evangelisti et al., 2017; Hayden et al., 2014; Meyer et al., 2016). As in other *Phytophthora* pathosystems (Evangelisti et al., 2017; Jupe et al., 2013), we observed up‐regulation of genes encoding candidate pathogenicity factors and MAMPs such as Hmp1, elicitins, necrosis and ethylene‐inducing proteins, glucanase and protease inhibitors, degradative enzymes (CAZymes, cutinases), RXLR effectors, and additional proposed effectors such as carbonic anhydrases. While we did identify expression of 12 candidate CRN proteins, none were predicted to possess signal peptides for secretion and thus are unlikely to be active effectors in *P. kernoviae*.

Invasion of plant tissues requires pathogens to breach physical barriers such as the cuticle, plant cell walls, and junctions between cells. Plant pathogens use enzymes such as cutinases, cellulases, and pectic enzymes to degrade these barriers (Kubicek et al., 2014; Nandi et al., 2018; Toth et al., 2003). Like other *Phytophthora* species (Brouwer et al., 2014; Ospina‐Giraldo et al., 2010), the *P. kernoviae* genome encodes many CAZymes, which may have a variety of roles in pathogen biology. We detected 211 of these within our RNA‐Seq experiment and found individual genes within the CAZyme families that exhibited strong up‐regulation during plant infection. However, because many CAZyme families were represented by multiple genes in *P. kernoviae*, we summed the expression values from each family to approximate the activity of each CAZyme family in each sample type. Ah Fong, Shrivastava, et al. (2017) used this approach to examine different CAZyme families in *P. infestans* and *Pythium ultimum* during infection of potato tuber tissue, revealing pathogen‐specific differences in expression levels for β‐1,3‐glucanase, β‐1,4‐glucanases, and pectate lyase.

Pectin‐degrading enzymes such as pectin/pectate lyase, pectinesterase, polygalacturonase, rhamnogalacturonase, and arabinan endo‐1,5‐α‐l‐arabinosidase were expressed at lower levels than cellulose or hemicellulose active CAZymes in *P. kernoviae*. A surprising finding was that, although *P. kernoviae* possesses and expresses multiple GH28 polygalacturonases that could degrade the homogalacturonan backbone, these enzymes were only expressed at low levels. Our data suggest that rhamnogalacturonan may be degraded before homogalacturonan in the early stages of infection, in conjunction with enzymes that digest the side chains. In *P. parasitica* infection of lupin, the pectin backbone may be degraded first, followed by side chains (Blackman et al., 2015).

As a tree pathogen, *P. kernoviae* may deploy lignin‐modifying enzymes such as laccases and lignin peroxidases to promote colonization of host tissues. While two laccases were found in the transcriptome, neither was expressed at detectable levels during infection. Lignin peroxidases fall within CAZy Auxiliary Activity family 2 (AA2). *P. kernoviae* expresses a single predicted extracellular AA2 peroxidase (TCONS_00004027.p1); another AA2 protein (TCONS_00002817.p3) is annotated as a mitochondrial protein. While TCONS_00004027.p1 is annotated as a catalase peroxidase, the predicted inclusion in family AA2 suggests it may function as a lignin peroxidase and will require further biochemical characterization to verify the substrate.

Like other *Phytophthora* species (McGowan & Fitzpatrick, 2017), the *P. kernoviae* genome encodes many candidate RXLR effectors. These may be translocated inside host cells to modify target proteins and promote infection, as they are in more extensively studied species such as *P. infestans* (Wang et al., 2017, 2018; Whisson et al., 2016). In our RNA‐Seq data, 69 putative *P. kernoviae* RXLR effectors were detectably expressed during infection of *N. benthamiana*. Because RXLR effectors often function to inhibit pattern‐triggered immunity (He et al., 2020), they are required from the earliest stages to facilitate infection. Consistent with this, we found that 48 of the *P. kernoviae* RXLR effectors, including eight of the top 10 highest expressed, exhibited greatest expression before disease symptoms were visible. Similarly in *P. infestans*, which possesses the largest number of predicted RXLR effectors (Haas et al., 2009; McGowan & Fitzpatrick, 2017; Thilliez et al., 2019), expression of up to 226 of the 563 predicted by Haas et al. (2009) was detected at 12 hpi, which is within the biotrophic phase of infection (Yin et al., 2017).

We used transient expression of 12 selected effectors in plant tissue, followed by pathogen infection, to gain a broad overview of whether only the most highly expressed RXLR effector‐coding genes led to increased pathogen colonization or if many more have the potential to contribute to pathogenicity. One RXLR effector resulted in cell death after infiltration, but nine of the remaining 11 effectors tested resulted in significantly increased lesion sizes. These were similar effects to those seen for these assays involving *P. infestans* effectors (e.g., Wang, McLellan, et al., 2019) in terms of the proportion that led to increased infection and the extent to which they increased infection.

Because RXLR effectors are known to be translocated into plant cells, the subcellular destinations of these have been determined by N‐terminal fusion to GFP or other fluorescent proteins. RXLR effectors from *P. infestans* and the downy mildew *Hyaloperonospora arabidopsidis* have shown localization to most cellular organelles and structures (with the possible exception of the actin cytoskeleton) (Caillaud et al., 2012; Wang, McLellan, et al., 2019). The 11 *P. kernoviae* RXLR effectors tested for their contribution to pathogenicity (above) localized to the cytoplasm, nucleus, nucleolus, and plasma membrane. This information is a valuable resource for interpreting outcomes from future experiments to identify the host proteins targeted by these effectors.

PkRXLR89 caused cell death in infiltrated leaves. Cell death phenotypes have also been observed for RXLR effectors from *P. parasitica*, *P. infestans*, and *P. agathidicida* (Guo et al., 2020; Huang et al., 2019; Wang, McLellan, et al., 2019). These effectors are either toxic when overexpressed in plant cells or are recognized by resistance proteins or other immune receptors, as for the PpE4 effector from *P. parasitica* or PaRXLR24 from *P. agathidicida* (Guo et al., 2020; Huang et al., 2019). PkRXLR89 is highly expressed during the biotrophic stage of *P. kernoviae* infection. If PkRXLR89 triggers an immune response through recognition by the host plant, then it is possible that another effector suppresses this cell death response, as for grapevine downy mildew *Plasmopara viticola* (Yin et al., 2019) and *P. agathidicida* (Guo et al., 2020). To date, RXLR effectors are the only oomycete effectors known to be recognized by host resistance proteins (Anderson et al., 2015). That *P. kernoviae* expresses RXLR effectors suggests that it may be possible to identify tree germplasm with genetic resistance to this pathogen, limiting the impact of this disease in forestry and informing (re)planting strategies to control disease.

## 4 EXPERIMENTAL PROCEDURES

### 4.1 *P*. *kernoviae* culture conditions

*P. kernoviae* (SCRP1055) was obtained from the *Phytophthora* culture collection at the James Hutton Institute, UK. Cultures were maintained on modified V8 juice agar (10% V8 juice, 1 g/L CaCO_3_, 2.5 mM KOH) and incubated in the dark at 20 °C.

### 4.2 RNA sequencing

The *P. kernoviae* transcriptome was analysed using RNA‐Seq of samples from cultured mycelium, spores, and three time points during infection of *N. benthamiana*. Spore samples were prepared by flooding 10‐day‐old V8 agar cultures of *P*. *kernoviae* with sterile distilled water, rubbing with a glass spreader rod, and gravity filtering through a 35 µm nylon filter. We found that *P. kernoviae* zoospores were released within the brief period needed to harvest sporangia and so a mixed spore sample (zoospores and sporangia) was prepared. The collected spores were centrifuged at 1,000 × g for 5 min, the supernatant discarded, then the recovered spores frozen in liquid nitrogen and stored at −70 °C until used for RNA isolation. Zoospores were used to inoculate 200 ml of amended lima bean medium (Bruck et al., 1981) and grown in the dark without shaking for 48 hr at 20 °C. Mycelium was harvested by filtration through 70 µm nylon mesh, excess liquid removed by blotting with sterile filter paper, then the sample was frozen in liquid nitrogen and stored at −70 °C until used for RNA extraction. For *N. benthamiana* infections, one leaf was taken from each of six plants (second fully expanded leaf) and placed in a clear plastic box on damp paper towels, abaxial side facing up. Each leaf was inoculated with four 20‐µl droplets of spores (5 × 10^4^ spores/ml), and the boxes sealed to maintain high humidity and incubated at 20 °C. Samples from each leaf were collected at 24, 48, and 72 hpi using a 5 mm diameter cork borer. Leaf disks were rinsed in sterile distilled water and blotted dry to remove any unbound and ungerminated spores, frozen in liquid nitrogen, and stored at −70 °C until used for RNA extraction. Four biological replicates were prepared for each sample.

RNA was extracted using a Qiagen RNeasy Plant Mini kit with the supplied protocol. RNA quality was assessed using a Bioanalyzer 2100 (Agilent) and spectrophotometry (NanoDrop). Sequencing libraries were prepared using a TruSeq RNA Library Preparation Kit v. 2 (Illumina), following the manufacturer's protocol. Libraries were quality‐controlled on a Bioanalyzer and sequenced (75 bp, paired‐end) on a NextSeq 500 (Illumina).

*P. kernoviae* genes were predicted using the MAKER pipeline v. 2.31.10 (Cantarel et al., 2008), incorporating ab initio gene prediction based on AUGUSTUS v. 3.1.0. This gene set was supplemented with RXLR effector‐coding genes curated manually for the presence of RXLR and EER motifs in the N‐terminal half of proteins, and those predicted by McGowan and Fitzpatrick (2017) (Table S10).

FastQC v. 0.11.5 (https://www.bioinformatics.babraham.ac.uk/projects/fastqc/), MultiQC v. 1.8 (Ewels et al., 2016), and Trimmomatic v. 0.39 (Bolger et al., 2014) were used for quality assessment of RNA‐Seq data. All filtered high‐quality reads were used for downstream analyses. The draft sequence of the *P. kernoviae* reference genome (strain 00238/432; Sambles et al., 2015; GenBank AOFI00000000) was used to align RNA‐Seq reads. Clean high‐quality reads were aligned to the reference genome using TopHat v. 2.0.12 and Cufflinks was used to assemble transcripts, both in default modes (Trapnell et al., 2012). TransDecoder‐v. 5.5.0 with default parameters was used to extract the most likely longest peptide‐coding candidate open reading frames (http://transdecoder.github.io) (File S1). Read mapping over assembled transcripts was used for expression quantification using FeatureCounts (Liao et al., 2014). Normalization and identification of DEGs used DESeq2 (Love et al., 2014) with the following criteria: (a) log_2_ fold‐change >0< and (b) false discovery rate (FDR) <0.05. Heatmap visualization of gene expression used Morpheus (https://software.broadinstitute.org/morpheus). The putative function of translated transcripts was explored through BLASTP similarity search against the Uniprot database with an e‐value cut‐off of 10^−10^. Secretory proteins were identified using SignalP v. 4.0 (Nordahl Petersen et al., 2011). Carbohydrate active proteins were predicted with dbCAN2 (Zhang et al., 2018) using HMMER (dbCAN HMMdb v9), DIAMOND, and Hotpep tools; the results were manually curated to remove mispredicted CAZy proteins.

### 4.3 Quantitative RT‐PCR analysis

RNA extraction, removal of DNA, first‐strand cDNA synthesis, and RT‐qPCRs were performed as described by Wang et al. (2018); primers are listed in Table S11. All assays were biologically replicated in triplicate and each technically replicated in triplicate; “no template” controls were included. Data analysis was based on that described in Avrova et al. (2003). The *P. kernoviae actinA* gene (TCONS_00001238.p1; GenBank KAF4323451) was used as an endogenous control as it exhibited less than 2‐fold variation in expression in RNA‐Seq and is homologous to the *ActA* gene used as an endogenous control for *P. infestans*.

### 4.4 Vector construction and *A. tumefaciens* transient assays

Candidate *P. kernoviae* RXLR effectors were cloned without their predicted signal peptides to retain them inside plant cells. Sequences were PCR amplified from *P. kernoviae* genomic DNA using gene‐specific primers that included Gateway recombination sites (Table S11), then introduced into pDONR201 using Gateway cloning (Invitrogen) to yield entry clones. Entry clones were recombined with destination vector pB7WGF2 (Karimi et al., 2005), containing the enhanced green fluorescent protein (eGFP) gene to yield N‐terminal GFP‐effector fusions, because the C‐terminus of RXLR effectors contains the functional domain. Destination vectors containing GFP‐effector sequences were electroporated into *A. tumefaciens* AGL1. Transformed *A. tumefaciens* strains were prepared for leaf infiltration as described in Wang, McLellan, et al. (2019).

ATTAs were carried out essentially as described by Kunjeti et al. (2016) and Wang, McLellan, et al. (2019). Leaves were kept on damp paper in sealed clear plastic boxes at 20 °C for 24 hr before *P. kernoviae* infection. *P. kernoviae* zoospores (10 µl, 5,000 zoospores/ml) were applied to each infiltration site. *P. kernoviae* is highly infective on *N. benthamiana*, thus we used a low number of zoospores per inoculation site in ATTAs to avoid an overwhelming inoculum load masking infection establishment effects that were specifically due to effector expression. Inoculated leaves were kept in sealed plastic boxes at 20 °C to maintain high humidity for disease development. Lesion diameter was measured at 4 dpi. Four sites were inoculated on each leaf and nine leaves were used for each of three replicates. A one‐way analysis of variance (ANOVA) Student–Newman–Keuls test was performed to identify statistically significant differences compared to the control (Wang, McLellan, et al., 2019).

### 4.5 Immunoblotting

Leaf disks (1 cm diameter) were harvested at 2 dpi after *Agrobacterium* infiltration with constructs expressing GFP‐RXLR effector fusion proteins. Leaf disks were ground in liquid nitrogen and resuspended in 100 µl of GTEN buffer (Boevink et al., 2016). Sample preparation was as described in Wang, McLellan, et al. (2019). Electrophoresis was performed as in Wang et al. (2018). Gel electroblotting, Ponceau staining, membrane blocking, and washing steps were carried out as described by McLellan et al. (2013). The αGFP primary antibody (Chromotek) was used at 1:2,000 dilution. Secondary antibody, anti‐mouse IgG horseradish peroxidase (Chromotek), was diluted 1:8,000. Immunoblotting membrane was processed with ECL substrate (Thermo Scientific Pierce) using the manufacturer's protocol and imaged with Amersham Hyperfilm ECL, developed with an Xograph imaging system, compact X4 developer.

### 4.6 Transformation of *P*. *kernoviae*

A previously published plasmid vector for expression of eGFP (pTor‐eGFP) in *Phytophthora* was used for transformation of *P. kernoviae* (Wang et al., 2017). *P. kernoviae* was transformed using the method described by Judelson et al. (1991); the full method can be found at https://oomyceteworld.net/Protoplast%20transformation.pdf. Transformed lines of *P. kernoviae* were selected on rye agar containing 10 µg/ml geneticin and later maintained on rye agar with 20 µg/ml geneticin.

### 4.7 Confocal imaging

*N. benthamiana* leaf cells transiently expressing effector fusions were observed at 24–48 hpi on a Nikon A1R confocal microscope, with images obtained using water‐dipping objectives. GFP was imaged using an excitation wavelength of 488 nm and the emissions were collected between 500 and 530 nm. mRFP and mOrange were excited with 561 nm light and emissions collected between 570 and 620 nm. The pinhole was set at 1.2 airy units for the longest wavelength fluorophore of any combination and coexpressed fluorophores were imaged sequentially to mimimize cross‐talk. Typically, cells expressing a low level of fluorescence were imaged to minimize overexpression artefacts. Each effector fusion was examined on multiple occasions on independent plants and in cells widely distributed across each infiltration zone to gauge the most typical fusion protein localization.

*N. benthamiana*, *Rhododendron*, and beech leaves infected with transgenic *P*. *kernoviae* expressing GFP were imaged using the confocal microscopy settings described above.

All images were processed with Nikon NIS Elements confocal software v. 4.30 and figures were compiled with Adobe Photoshop.

## Supporting information


**FIGURE S1** (a) Principal component analysis (PCA) of replicated samples used for RNA sequencing (RNA‐Seq), showing clear separation of mycelium (Myc), spore (Spr), and infection samples (T24, T48, T72). PCA was performed on the sample correlation matrix calculated from log_2_‐transformed normalized expression count values from DESeq2. (b) Hierarchical clustering (one minus Pearson correlation) of all *Phytophthora kernoviae* RNA‐Seq samples in matrix format; all genes were used for clustering. Red shading denotes close similarity and blue denotes dissimilarity. (c) Volcano plot of the *P. kernoviae* transcriptome showing distribution of differentially expressed genes (DEGs) in the different samples compared to cultured mycelium

**FIGURE S2** Heatmap of differentially expressed *Phytophthora* *kernoviae* carbohydrate active protein (CAZy) genes from RNA sequencing in cultured mycelium (Myc), spores (mixture of sporangia and zoospores; Spr), and infection of *Nicotiana benthamiana* at 24, 48, and 72 hr postinoculation (hpi) (T24, T48, T72). Red shading indicates increased transcript levels and blue shading indicates lower transcript levels. Colour shading represents expression values transformed by subtracting the mean and dividing by the standard deviation for each row (gene). (a) CAZymes grouped by family. (b) CAZymes clustered (Pearson hierarchical clustering) according to expression pattern

**FIGURE S3** Expression of selected *Phytophthora* *kernoviae* RXLR effector coding genes. This figure shows two additional independent biological replications (a–b, c–d) of the quantitative reverse transcription‐PCR experiment shown in Figure 3. Transcript levels are shown relative to that in cultured mycelium, which was normalized to a value of 1. Error bars shown are standard error

**FIGURE S4** Stability of GFP‐PkRXLR effector fusions. Proteins were extracted from *Agrobacterium tumefaciens* infiltrated *Nicotiana benthamiana* leaves and the blotted proteins were probed with mouse anti‐GFP antibody (αGFP). All of the tested *Phytophthora* *kernoviae* RXLR effector fusions show a protein band at the expected size, indicated by white asterisks. Size markers (kilodaltons) are indicated at the right of the western blot. Protein loading is shown below the western blot as Ponceau stain (PS)

**FIGURE S5** Characterization of nuclear localized *Phytophthora* *kernoviae* effectors. (a) PkRXLR79 and PkRXLR97 both localize to the nucleoplasm and nucleolus, but PkRXLR79 is most abundant at the periphery of the nucleolus. In contrast, a coexpressed mRFP‐histone 2B (mRFP‐H2B) fusion labels only the nucleoplasm. (b) PkRXLR99 localizes to the nucleoplasm and nucleolus, and is also most abundant at the periphery of the nucleolus. However, PkRXLR99 also forms aggregates in the nucleus, separate from the nucleolus, and from which the mRFP‐H2B is excluded. Coexpression of PkRXLR99 with mRFP‐fibrillarin (mRFP‐Fib1) was used to show the aggregated PkRXLR99 is separate to the nucleolus. Scale bar represents 10 µm

**FIGURE S6** Characterization of plasma membrane localized *Phytophthora* *kernoviae* effectors. Coexpression of PkRXLR132 and PkRXLR123 with a marker for the plant plasma membrane, mOrange‐LTi6b. PkRXLR132 localizes only to the plasma membrane, while PkRXLR123 also localizes to the nucleus and nucleolus. Scale bars represent 50 µm

**TABLE S1** Sample names and Illumina NextSeq sequence output for *Phytophthora kernoviae* RNA sequencing experiment

**TABLE S2** Normalized expression count (from DESeq2) from RNA sequencing for *Phytophthora kernoviae* in cultured mycelium (Myc), mixed asexual spores (Spr), and infection of *Nicotiana benthamiana* at 24, 48, and 72 hr postinfection (T24, T48, T72)

**TABLE S3** Pairwise differentially expressed *Phytophthora kernoviae* transcripts from RNA sequencing (DESeq2 analysis); up‐regulated transcripts

**TABLE S4** Pairwise differentially expressed *Phytophthora kernoviae* transcripts from RNA sequencing (DESeq2 analysis); down‐regulated transcripts

**TABLE S5** Normalized expression count values for *Phytophthora kernoviae* RXLR effector coding genes

**TABLE S6** Transcript abundance for *Phytophthora kernoviae* CAZyme coding genes

**TABLE S7** Summed mean normalized expression count values for *Phytophthora kernoviae* carbohydrate active enzyme families (CAZymes)

**TABLE S8** Normalized expression count values for selected *Phytophthora kernoviae* apoplastic effectors and PAMP coding genes

**TABLE S9** Normalized expression count values for *Phytophthora kernoviae* crinkle and necrosis (CRN) effector coding genes

**TABLE S10** Candidate RXLR proteins from *Phytophthora kernoviae*

**TABLE S11** Oligonucleotide primers used for making plasmid constructs for transient expression in *Nicotiana benthamiana* and quantitative reverse transcription PCR

**FILE S1** Fasta file of *Phytophthora kernoviae* proteins predicted by Transdecoder

## Data Availability

Illumina sequence data were deposited in the NCBI Sequence Read Archive at https://www.ncbi.nlm.nih.gov/sra under BioProject PRJNA506645, accessions from SRX5057214 to SRX5057233. MAKER parameter values and settings can be found in the MAKER configuration files at FigShare (https://doi.org/10.6084/m9.figshare.2168530). The resulting annotations are available in EnsemblProtists at http://protists.ensembl.org/Phytophthora_kernoviae. Raw output files are also posted in this same FigShare project. MAKER annotations were submitted to GenBank via the NCBI web portal and are available under BioProject (https://www.ncbi.nlm.nih.gov/bioproject) accesion number PRJNA184368 and BioSample (https://www.ncbi.nlm.nih.gov/biosample) accession number SAMN02981496.
