# Supplementary material for: Haustorium formation and a distinct biotrophic transcriptome characterize infection of Nicotiana benthamiana by the tree pathogen Phytophthora kernoviae
Source: Mol Plant Pathol. 2021 May 20;22(8):954–68. doi: 10.1111/mpp.13072 (PMC8295517; doi:10.1111/mpp.13072)
Supplement: Supplementary file 3 — FIGURE S3 Expression of selected Phytophthora kernoviae RXLR effector coding genes. This figure shows two additional independent biological replications (a–b, c–d) of the quantitative reverse transcription‐PCR experiment shown in Figure 3. Transcript levels are shown relative to that in cultured mycelium, which was normalized to a value of 1. Error bars shown are standard error [file MPP-22-954-s011.pdf]

a

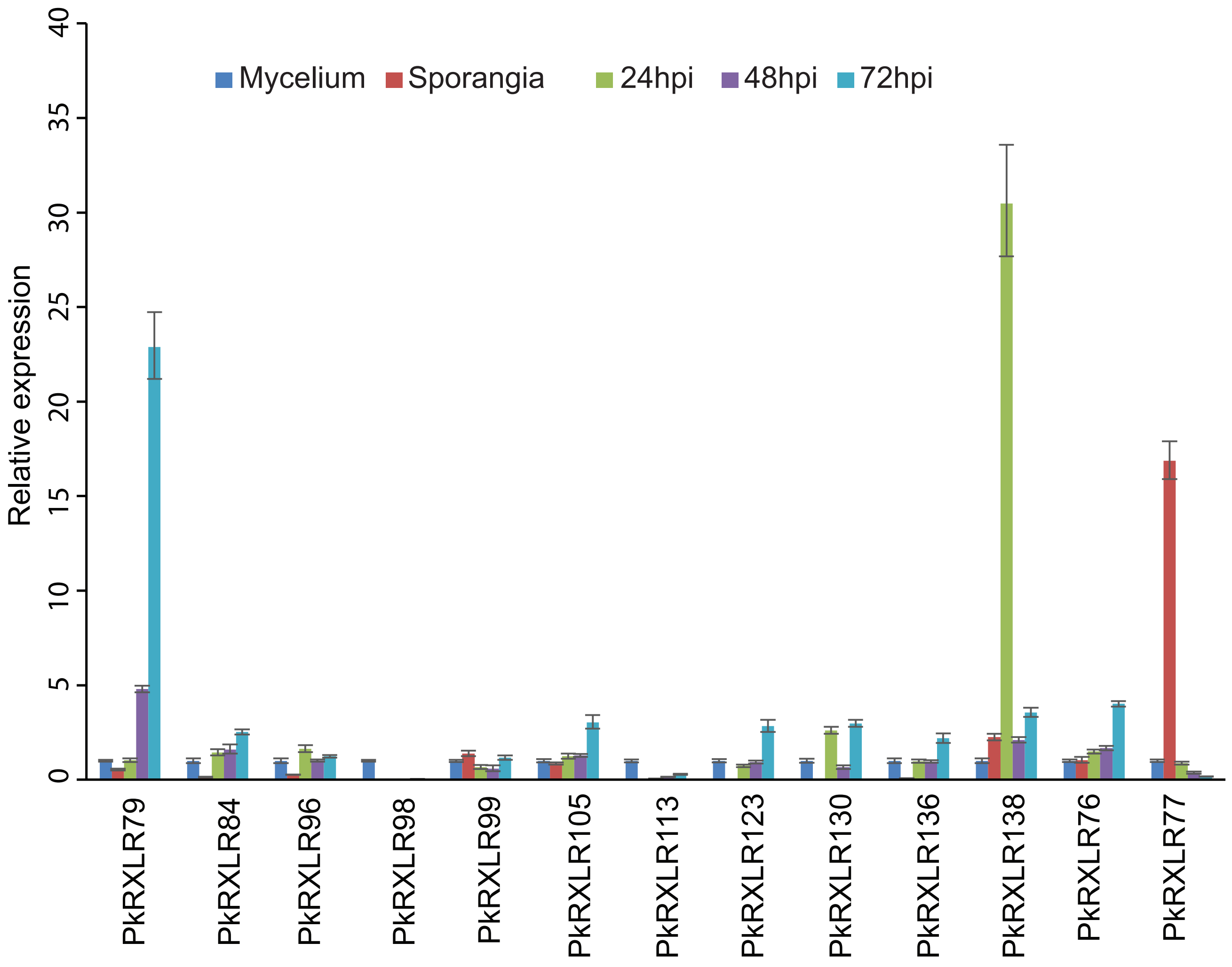

b

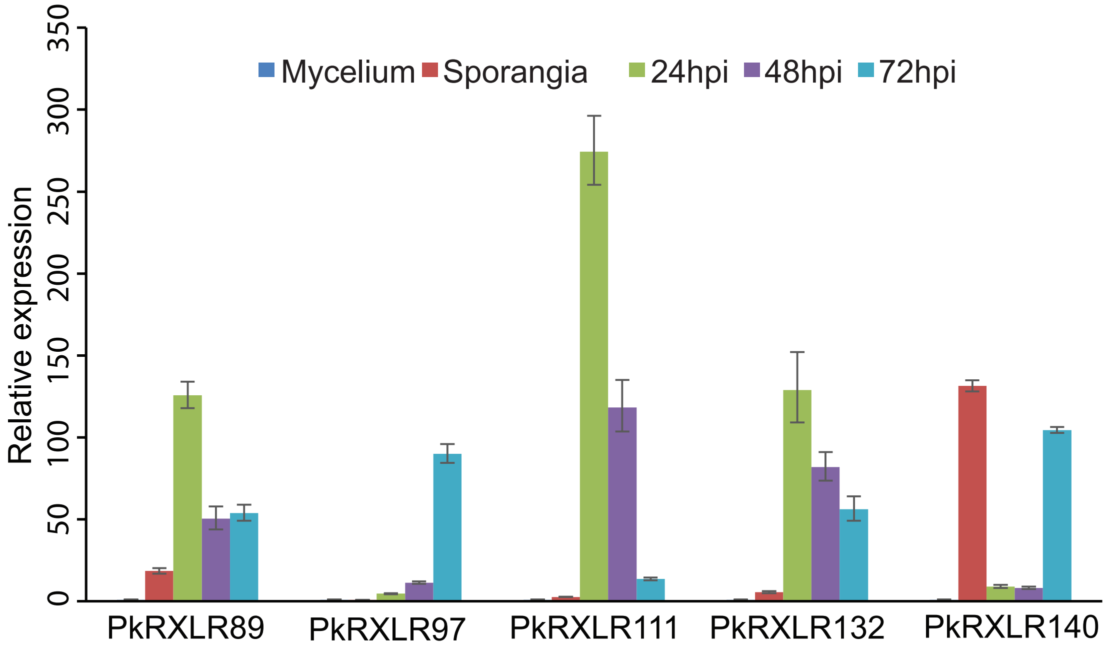

Supplementary Figure S3: Expression of selected *P. kernoviae* RXLR effector coding genes. This figure shows two additional independent biological replications (a-b, c-d [next page]) of the quantitative RT-PCR experiment shown in Figure 3. Transcript levels are shown relative to that in cultured mycelium, which was normalized to a value of one. Error bars shown are standard error.

c

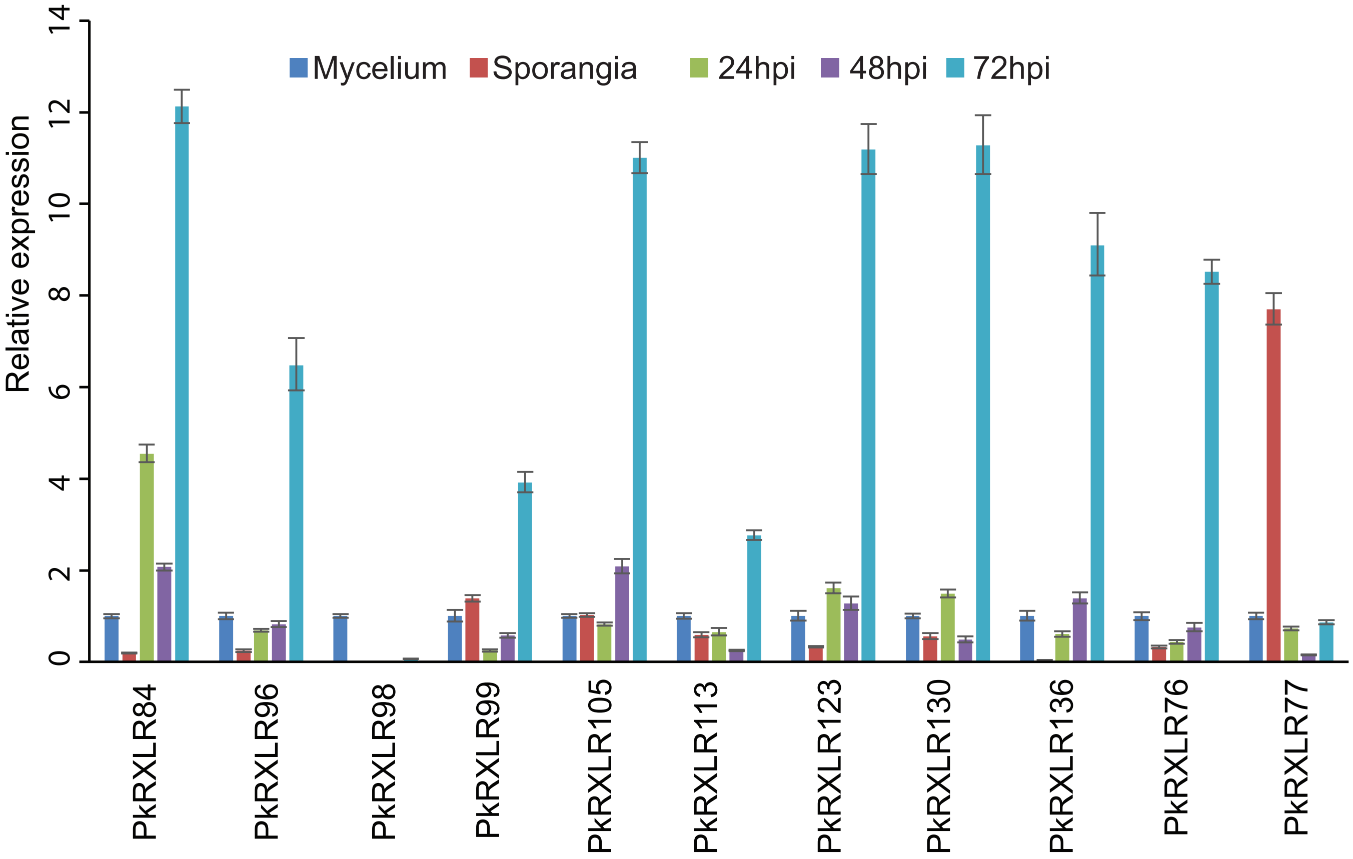
Mycelium
Sporangia
24hpi
48hpi
72hpi
Relative expression
0
2
4
6
8
10
12
14
PkRXLR84
PkRXLR96
PkRXLR98
PkRXLR99
PkRXLR105
PkRXLR113
PkRXLR123
PkRXLR130
PkRXLR136
PkRXLR76
PkRXLR77

d

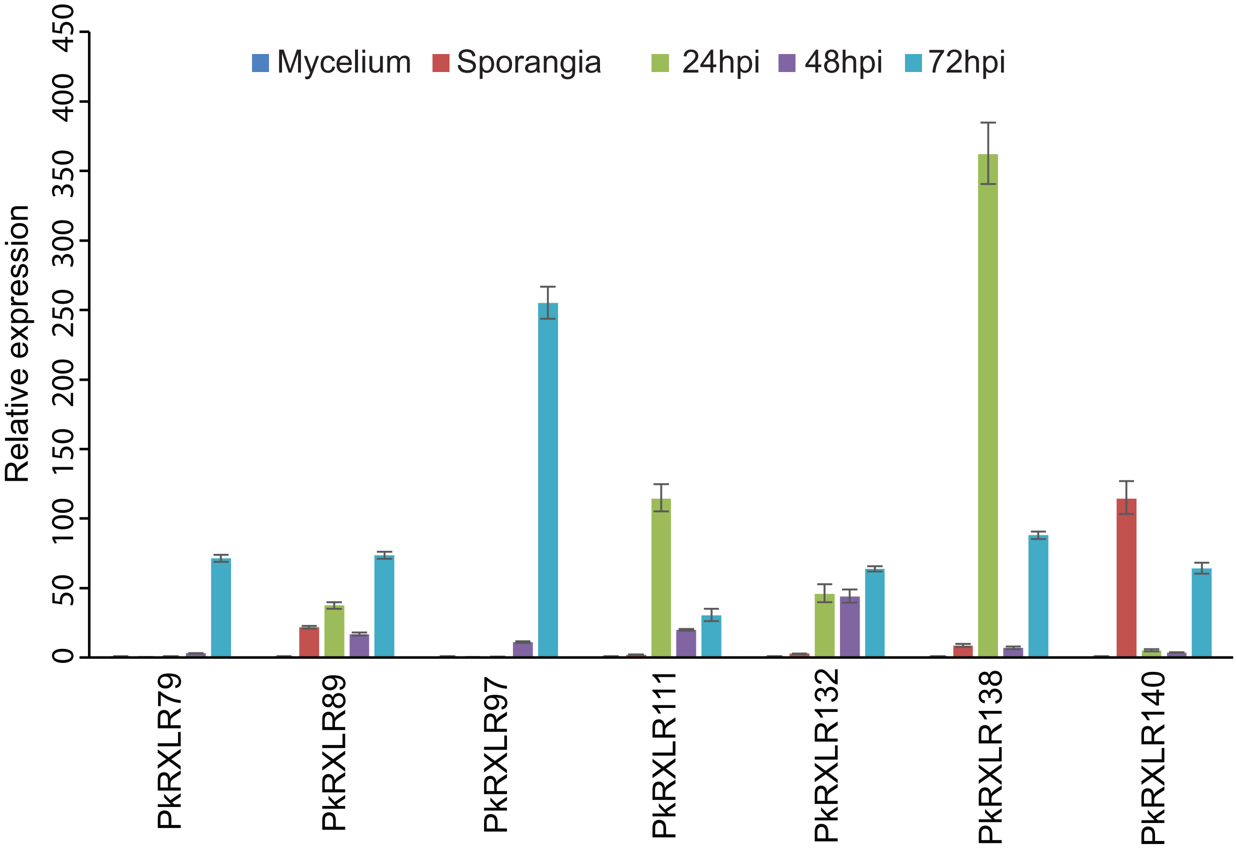
Mycelium
Sporangia
24hpi
48hpi
72hpi
Relative expression
0
50
100
150
200
250
300
350
400
450
PkRXLR79
PkRXLR89
PkRXLR97
PkRXLR111
PkRXLR132
PkRXLR138
PkRXLR140
