# Supplementary figures and images for: Haustorium formation and a distinct biotrophic transcriptome characterize infection of Nicotiana benthamiana by the tree pathogen Phytophthora kernoviae
Source: Mol Plant Pathol. 2021 May 20;22(8):954–68. doi: 10.1111/mpp.13072 (PMC8295517; doi:10.1111/mpp.13072)

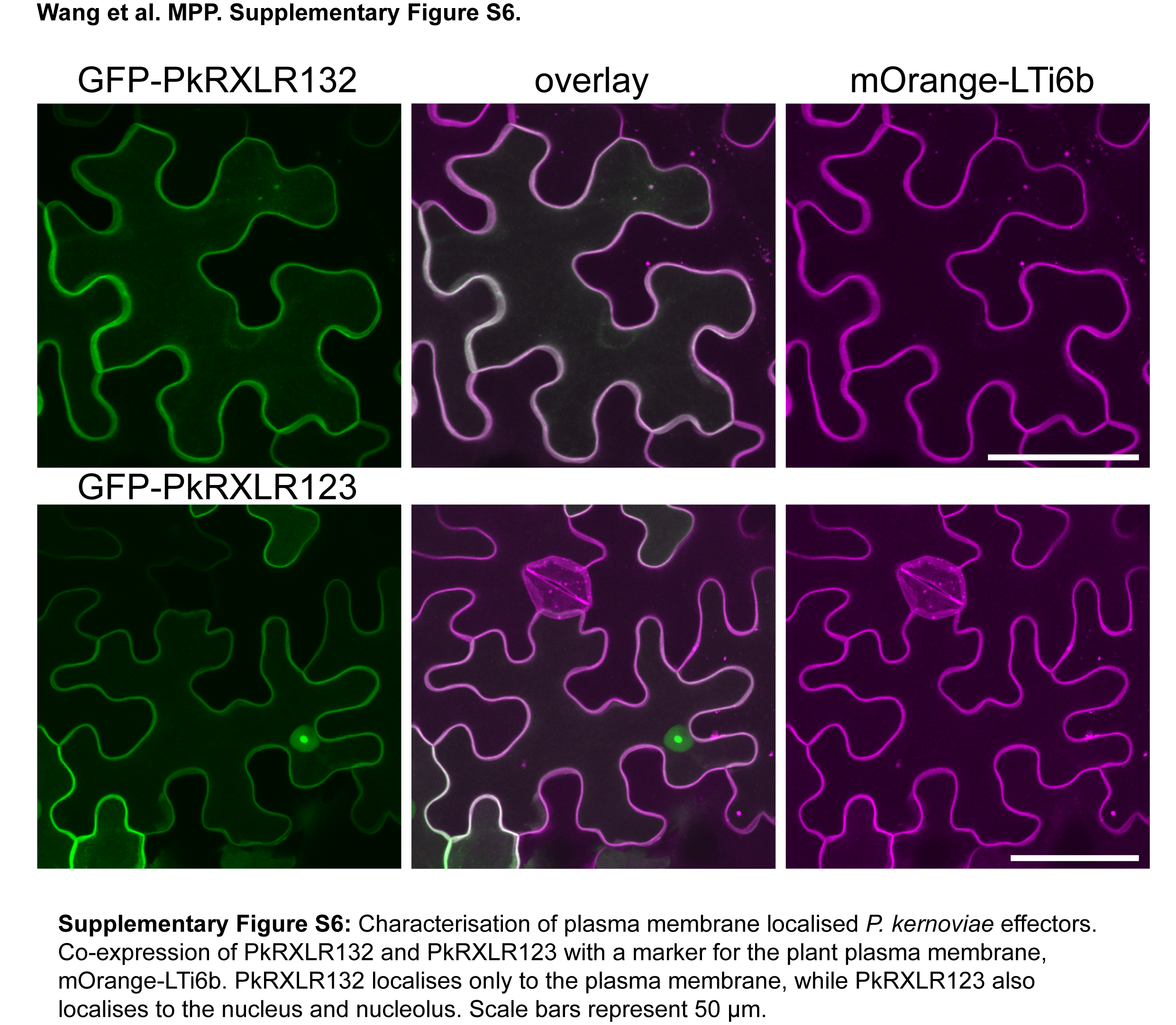

Supplement: Supplementary file 6 — FIGURE S6 Characterization of plasma membrane localized Phytophthora kernoviae effectors. Coexpression of PkRXLR132 and PkRXLR123 with a marker for the plant plasma membrane, mOrange‐LTi6b. PkRXLR132 localizes only to the plasma membrane, while PkRXLR123 also localizes to the nucleus and nucleolus. Scale bars represent 50 µm [file MPP-22-954-s010.tif]
